# Intracluster correlation coefficients in a large cluster randomized vaccine trial in schools: Transmission and impact of shared characteristics

**DOI:** 10.1371/journal.pone.0254330

**Published:** 2021-10-14

**Authors:** Jane Whelan, Helen Marshall, Thomas R. Sullivan

**Affiliations:** 1 Clinical and Epidemiology Research and Development, GlaxoSmithKline Vaccines B.V., Amsterdam, The Netherlands; 2 Vaccinology and Immunology Research Trials Unit, Women’s and Children’s Health Network, Adelaide, South Australia, Australia; 3 Robinson Research Institute and Adelaide Medical School, The University of Adelaide, Adelaide, South Australia, Australia; 4 SAHMRI Women & Kids, South Australian Health & Medical Research Institute, Adelaide, Australia; 5 School of Public Health, University of Adelaide, Adelaide, South Australia, Australia; UCLA, UNITED STATES

## Abstract

Cluster randomized trials (cRCT) to assess vaccine effectiveness incorporate indirect effects of vaccination, helping to inform vaccination policy. To calculate the sample size for a cRCT, an estimate of the intracluster correlation coefficient (ICC) is required. For infectious diseases, shared characteristics and social mixing behaviours may increase susceptibility and exposure, promote transmission and be a source of clustering. We present ICCs from a school-based cRCT assessing the effectiveness of a meningococcal B vaccine (Bexsero, GlaxoSmithKline) on reducing oropharyngeal carriage of *Neisseria meningitidis* (*Nm*) in 34,489 adolescents from 237 schools in South Australia in 2017/2018. We also explore the contribution of shared behaviours and characteristics to these ICCs. The ICC for carriage of disease-causing *Nm* genogroups (primary outcome) pre-vaccination was 0.004 (95% CI: 0.002, 0.007) and for all *Nm* was 0.007 (95%CI: 0.004, 0.011). Adjustment for social behaviours and personal characteristics reduced the ICC for carriage of disease-causing and all *Nm* genogroups by 25% (to 0.003) and 43% (to 0.004), respectively. ICCs are also reported for risk factors here, which may be outcomes in future research. Higher ICCs were observed for susceptibility and/or exposure variables related to *Nm* carriage (having a cold, spending ≥1 night out socializing or kissing ≥1 person in the previous week). In metropolitan areas, nights out socializing was a highly correlated behaviour. By contrast, smoking was a highly correlated behaviour in rural areas. A practical example to inform future cRCT sample size estimates is provided.

## Introduction

Controlled trials, randomized at the individual level, have been the mainstay of vaccine efficacy trials, particularly for licensure. However, due to the nature of the intervention or for logistical or other reasons, it is not always practical to individually randomize participants [1, 2]. In trials assessing vaccine effectiveness, the overall effect of vaccination incorporating indirect effects may be more important than the direct effect alone to inform public health and vaccination policy [3]. The cluster randomized controlled trial (cRCT) design captures total or overall vaccine effects, offering an advantage in this regard.

A key feature of cRCTs to evaluate infectious disease interventions (in contrast to studies evaluating community health promotion interventions, for example), is that cases within clusters can transmit infection to other cluster members. Individuals within clusters may share similar behaviours, as well as characteristics, that make them more susceptible to infection, but these shared behaviours within the same contact network may also predict social mixing (with more or less interpersonal distance), and therefore increased exposure to infection. *Neisseria meningitidis* (*Nm)*, for example, is transmitted via respiratory and salivary secretions and requires close contact for transmission. Consequently, social behaviours (e.g. socializing in bars, kissing) and individual behaviours (e.g. smoking), as well as fixed personal characteristics (adolescence/ young adulthood and male sex [4]), may lead to increased susceptibility and / or exposure and are associated with increased *Nm* pharyngeal carriage prevalence [5] and, more rarely, with invasive meningococcal disease [6]. Intracluster similarities, therefore, lead to individual outcomes that are correlated within clusters, rather than independent. Due to this correlation, often quantified as the intracluster correlation coefficient (ICC), the cRCT design requires a larger sample size to estimate treatment effects with the same degree of precision as an individual randomized controlled trial [7].

Despite its importance to the design of cRCTs assessing infectious disease interventions, the ICC is rarely known with any certainty in advance. Assumptions made by researchers regarding the ICC *a priori* are increasingly reported, and estimates are derived often with reference to the literature (as was the case for the ICC assumed for the cRCT for which a post-hoc estimate is derived from the study data, reported here), or occasionally informed by pre-existing data [8, 9] or baseline analyses [10, 11]. As recommended within the ‘CONSORT statement: extension to cluster randomized trials’, here we report the ICCs obtained from a large school-based cRCT assessing the impact of a meningococcal B (MenB) vaccine (Bexsero, GSK) on pharyngeal carriage of *Nm* in adolescents in schools in South Australia (SA) in 2017/2018. While it is widely recognized that adjustment for covariates in a model will reduce the estimated ICC [12], this study aimed to explore the impact of fixed characteristics (e.g. such as age, race/ethnicity, urban versus rural location) and social behaviours known to increase carriage prevalence and the extent to which they are also correlated within clusters, reflecting the degree of social mixing. As well as providing ICCs across all schools, we separately report ICCs for schools in metropolitan and rural areas where there were some differences in *Nm* carriage prevalence and where we expected personal behaviours and social mixing patterns might also differ. We also provide ICCs for the behavioural factors to give context to their effects on carriage ICCs and as they may be relevant for outcomes of future research (smoking and the experience of respiratory symptoms are outcomes in previous cluster randomized trials [13, 14], and questions arise—most recently in relation to SARS-CoV-2, for example—on the impact of public health guidance and government advice on social gatherings and behavior). Finally, in the context of pragmatic or pseudo-interventional community trial design, where epidemiologists and public health practitioners may be involved in study design as well as clinical trailists and statisticians, we provide a practical example on the use of ICCs to inform sample size estimates for future cRCTs.

## Materials and methods

This study involved a post-hoc analysis of data that were collected for a school-based, cluster randomized controlled trial [15, 16]. The cRCT was approved by the Women’s and Children’s Health Network Human Research Ethics Committee, for which informed consent was obtained in writing. The study was conducted in SA in 2017 to assess the impact of a meningococcal B vaccine (Bexsero) on pharyngeal carriage of *Nm* in school-going adolescents (NCT03089086). SA has a total population of 1.72 million and each school year level comprises around 19,000–20,000 students. Students in years 10–12 (aged 15–18 years) in all 260 high schools in SA were invited to participate in a cRCT and schools were randomized to MenB vaccination at baseline (intervention) or at 12 months (control). At baseline and at 12 months, participants completed a questionnaire on pre-disposing personal characteristics (age, sex, ethnicity) and variables related to susceptibility and / or exposure to pharyngeal carriage of *Nm* (social mixing, household size, smoking history, recent antibiotic use and upper respiratory tract infection). A copy of the questionnaire (S1 Fig) and a full list of the variables and how they were defined (S1 Table is provided in the supporting information). Schools were classified as metropolitan or rural based on the Index of Community Socio-educational Advantage (ICSEA) classification [17]. An oropharyngeal swab was collected from each participant. The primary outcome was oropharyngeal carriage of disease-causing *Nm* (groups A,B,C,W,X,Y), identified by both porA and genogroup PCR assays in year 10/11 students at 12 months [16]. Secondary outcomes included carriage of ‘all *Nm*’ (capsulated and non-groupable) and acquisition of *Nm* from non-carrier to carrier status. Risk factors for carriage were also assessed at baseline. During the study design phase, it was estimated that a sample size of 12,160 year 10/11 students per group would allow for the detection of a 20% relative reduction in carriage of disease-causing *Nm* with 90% power (two-tailed α = 0.05), from an assumed prevalence of 8% among the unvaccinated at 12 months. To account for the cRCT design, a design effect of 2.19 was incorporated based on an expected average of 120 year 10/11 students per school, and a conservative ICC estimate of 0.01, which was derived from the literature [18] in the absence of pilot data or estimates based on *Nm* transmission.

On trial completion, ICCs for carriage of disease-causing and overall *Nm*, and known risk factors for carriage were estimated in separate logistic regression models, with the variable of interest treated as an outcome in the logistic model and using generalized estimating equations to account for clustering within schools. For each variable, the ICC was taken to be the estimated correlation parameter of the exchangeable working correlation structure [19]. This approach provides an estimate of the ICC on the proportion scale, which is consistent with several other ICC estimators but not random effects logistic regression, whose estimate is on the logistic scale. To explore the degree to which ICCs for carriage measures were influenced by social behaviours and shared characteristics, the logistic models for carriage were fitted with and without adjustment for these factors. Additionally, for the 12-month carriage outcomes, an adjustment was made for treatment group, as ICCs ignoring potential treatment effects can be biased [20]. 95% confidence interval (CI) limits around the ICCs were constructed using 2000 bootstrap samples of the participating schools and computing a biased-corrected interval. All statistical calculations were performed using Stata version 15.0 (Stata Corp., College Station, TX).

## Results

**Fig 1:**
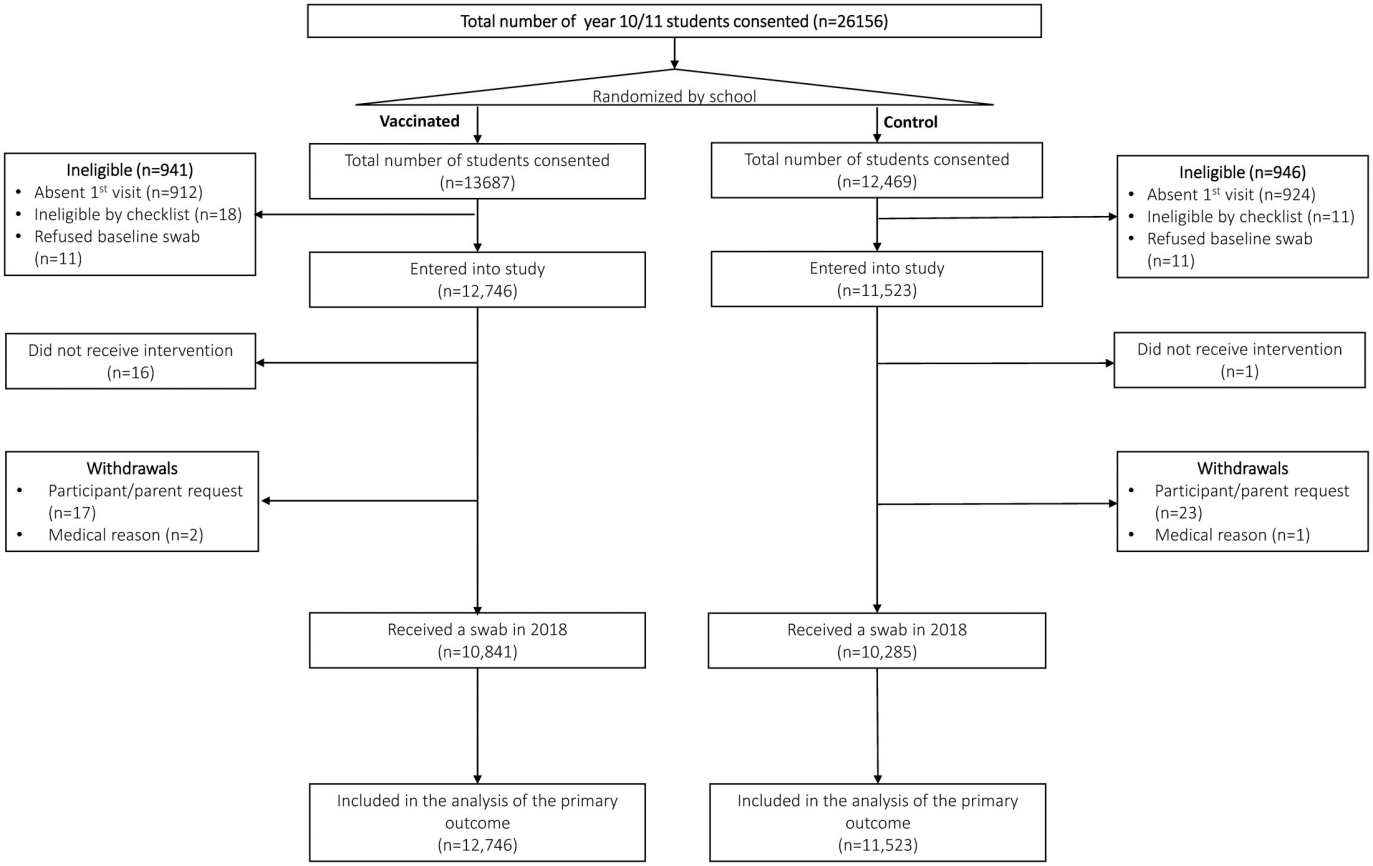
Flow chart for inclusion of schools into trial.

In 2017 and 2018, 237 of 260 secondary schools throughout the state of SA enrolled in the cRCT (124 metropolitan and 113 rural schools). The study cohort comprised 34,489 secondary school students, of whom 24,269 year 10/11 students contributed to the primary objective (Fig 1).

At baseline, the median number of year 10/11 students per cluster was 83 (interquartile range (IQR): 22 to 161). At 12 months, swab data on 21,126 year 10/11 students were collected across 230 schools, with a median cluster size of 72.5 (IQR: 20 to 142). Demographic characteristics and risk factors at baseline are described in Table 1.

**Table 1:** Baseline characteristics of participants (school years 10 to 12) in a cluster randomized controlled trial to assess oropharyngeal carriage of *Neisseria meningitidis* by location in South Australia in 2017/2018.

Characteristic	Overall (n = 34,489)	Metropolitan (n = 25,579)	Rural (n = 8,910)
	n (%)	n (%)	n (%)
Vaccine intervention group	18,362 (53.2)	14,183 (55.5)	41,79 (46.9)
Year of schooling			
10	12,764 (37.0)	9,329 (36.5)	3,435 (38.6)
11	11,505 (33.4)	8,647 (33.8)	2,858 (32.1)
12/13	10,220 (29.6)	7,603 (29.7)	2,617 (29.4)
Age—years: mean (standard deviation)	16.05 (1.1)	16.06 (1.2)	16.00 (1.0)
School size (No. of students per year)			
<60 students/year (small)	5,298 (15.4)	26,24 (10.3)	2,674 (30.0)
60 to 119 students/year (medium)	11,521 (33.4)	8,576 (33.5)	2,945 (33.1)
>119 students/year (large)	17,670 (51.2)	14,379 (56.2)	3,291 (37.0)
Female	17,921 (52.0)	13,266 (51.9)	4,655 (52.2)
Ethnicity			
White	24,701 (72.91)	17,963 (71.3)	6,738 (77.5)
Aboriginal/Torres Strait Islander	941 (2.878)	516 (2.1)	425 (4.9)
Asian	3,383 (10.09.99)	3,189 (12.7)	194 (2.2)
Other	4,853 (14.32)	3,518 (14.0)	1,335 (15.4)
Current cold or sore throat	7,218 (21.11)	5,502 (21.7)	1,716 (19.5)
Smoked cigarettes in the last week	628 (1.83)	406 (1.6)	222 (2.5)
Smoked water-pipe in last week	1,042 (3.105)	836 (3.3)	206 (2.3)
Out one or more days in last week	7,067 (20.60)	5,087 (20.0)	1,980 (22.3)
Kissed one or more people in last week	7,753 (23.105)	5,549 (22.2)	2,204 (25.5)
Disease-causing carriage (genogroups ABCWXY)	668 (1.94)	491 (1.9)	177 (2.0)
Overall carriage	1,222 (3.655)	861 (3.4)	361 (4.1)

The most prevalent behaviours at baseline included one or more days out in the last week (20.6%), having a current cold or sore throat (21.1%), and intimately kissing one or more persons in the last week (23.1%). In a multivariable logistic model using generalized estimating equations, as reported in Marshall et al [16], statistically significant associations were observed between baseline carriage of disease-causing *Nm* genogroups and year of schooling (adjusted odds ratio (aOR) year 12 vs 10 = 2.75; 95% CI:2.03–3.73, p < .0001), current cold or sore throat (aOR = 1.35; 95% CI:1.12–1.63, p = 0.002), smoking cigarettes (aOR = 1.91; 95% CI:1.29–2.83, p = 0.001); smoking a water-pipe (aOR = 1.82; 95% CI:1.30–2.54, p = 0.0005), attending pubs/clubs (aOR = 1.54; 95% CI:1.28–1.86, p = < .0001); and intimate kissing (aOR = 1.65; 95% CI:1.33–2.05, p = < .0001). (Of note, the carriage prevalence of disease-causing *Nm* at 12 months (primary outcome) was 2.55% (326/12,746) among the vaccinated group and 2.52% (291/11,523) among controls [16]).

At baseline, the ICC for carriage of disease-causing *Nm* genogroups was 0.004 overall (95% CI:0.002–0.007) (Table 2). The ICCs for days out, kissing and having a cold or sore throat in the last week were many magnitudes higher (8.5, 3.75 and 5.25 times higher, respectively) than that of the ICC observed for baseline carriage of disease-causing genogroups, which is not surprising given that more prevalent characteristics are typically associated with higher ICCs [21]. Adjustment for these risk factors had limited impact on the baseline ICC estimate for carriage of disease-causing genogroups (ICC = 0.003; 95% CI:0.002–0.005) but reduced the ICC point estimate for all *Nm* carriage by 43% from 0.007 to 0.004. At 12 months, the ICC for carriage of disease-causing genogroups adjusted only for treatment group was 0.006 (95% CI:0.003–0.010), with additional adjustment for behavioural risk factors again having minor impact (adjusted ICC = 0.005; 95% CI:0.002–0.009). The ICC point estimate for all *Nm* carriage at 12 months was reduced on adjustment for risk factors from 0.008 to 0.006.

**Table 2:** Intracluster correlation coefficients for *Nm* carriage and behavioural risk factors, overall and by location at baseline and at 12 months.

Variable	Overall (95% CI)	Metropolitan (95% CI)	Rural (95% CI)
*1*. *Baseline carriage*			
Disease-causing carriage	0.004 (0.002, 0.007)	0.004 (0.002, 0.007)	0.006 (0.000, 0.014)
Overall carriage	0.007 (0.004, 0.011)	0.006 (0.003, 0.011)	0.014 (0.006, 0.025)
*2*. *Baseline behavioural risk factors*			
Current cold or sore throat	0.021 (0.015, 0.031)	0.021 (0.015, 0.032)	0.016 (0.010, 0.026)
Smoked cigarettes in last week	0.008 (0.005, 0.013)	0.005 (0.003, 0.007)	0.020 (0.011, 0.035)
Smoked water-pipes in last week	0.007 (0.005, 0.010)	0.006 (0.004, 0.009)	0.010 (0.006, 0.015)
Days out in last week (0 vs. 1 or more)	0.034 (0.022, 0.049)	0.041 (0.026, 0.062)	0.015 (0.007, 0.027)
People kissed in last week (0 vs. 1 or more)	0.015 (0.011, 0.020)	0.015 (0.010, 0.020)	0.010 (0.005, 0.019)
*3*. *Baseline carriage*, *adjusted for risk factors* ^a^			
Disease-causing carriage	0.003 (0.001, 0.005)	0.003 (0.001, 0.004)	0.006 (0.000, 0.014)
Overall carriage	0.004 (0.002, 0.006)	0.003 (0.002, 0.005)	0.009 (0.005, 0.016)
*4*. *Carriage outcomes at 12 months (year 10*, *11 students only)* ^b^			
Disease-causing carriage ^c^	0.006 (0.003, 0.010)	0.005 (0.002, 0.011)	0.005 (0.001, 0.010)
Disease-causing carriage, adjusted for baseline risk factors ^a^	0.005 (0.002, 0.009)	0.005 (0.002, 0.010)	0.005 (0.001, 0.012)
Overall carriage ^c^	0.008 (0.004, 0.013)	0.007 (0.003, 0.013)	0.007 (0.001, 0.016)
Overall carriage, adjusted for baseline risk factors ^a^	0.006 (0.003, 0.011)	0.005 (0.002, 0.011)	0.004 (0.000, 0.010)
Acquisition disease-causing carriage ^c^	0.005 (0.002, 0.010)	0.005 (0.002, 0.011)	0.004 (0.000, 0.009)
Acquisition disease-causing carriage, adjusted for baseline risk factors ^c^	0.005 (0.002, 0.009)	0.005 (0.001, 0.009)	0.004 (0.000, 0.012)
Acquisition overall carriage ^c^	0.007 (0.003, 0.012)	0.007 (0.003, 0.014)	0.003 (0.000, 0.009)
Acquisition overall carriage, adjusted for baseline risk factors ^a^	0.006 (0.003, 0.010)	0.006 (0.002, 0.011)	0.002 (0.000, 0.009)

^a^ Includes all behavioural risk factors in (2), plus year of schooling (10 or 11), ethnicity (White, Asian, Aboriginal or Torres Strait Islander, Other) and baseline disease-causing or overall *Nm* carriage (for disease-causing and overall *Nm* carriage outcomes at 12 months only).

^b^ All Intracluster correlation coefficients (ICCs) for disease outcomes at 12 months were adjusted for randomized group.

^c^ The primary outcome of the cRCT was the carriage prevalence at 12 months in the vaccine vs. control groups, 2.55% vs. 2.52% respectively.

In metropolitan versus rural areas, baseline carriage prevalence of disease-causing *Nm* genogroups was 1.92% and 1.99%, respectively and the ICC point estimate was 0.004 (metro) and 0.006 (rural). For all *Nm* genogroups, baseline prevalence was 3.37% (metro) and 4.96% (rural). The point estimate for the ICC was 2.3 times higher in rural areas versus metropolitan at baseline (0.014 versus 0.006, Table 2). Cigarette smoking was more prevalent and a more correlated behavior in rural versus metropolitan schools (0.020 [rural] vs 0.005 [metro]). By contrast, the ICC associated with nights out socializing was almost 3 times higher in metropolitan versus rural schools (0.041 vs 0.015, respectively), despite the reported behavior being similarly prevalent in each setting (20% in metro schools versus 22.3% in rural schools). At 12 months, the prevalence of disease-causing *Nm* genogroups was 2.3% versus 3.2% in metropolitan and rural areas respectively, and both adjusted and unadjusted ICCs were similar. Prevalence of all *Nm* was 4.0% versus 6.1% (Table S4 in Marshall et al [16]) and adjustment reduced the ICCs by 29% (metro) and 43% (rural).

### Example sample size calculation

To illustrate how the ICCs in Table 2 can inform sample size calculations, consider a hypothetical trial comparing a new meningococcal vaccine versus control in a school-based cRCT involving adolescents. The primary outcome will be carriage at 12 months, with a 25% relative reduction from an assumed control prevalence of 6% deemed to be the smallest clinically important effect worth detecting. Assuming independent outcomes, 4,644 students would be required to have 90% power to detect a reduction in overall carriage from 6% to 4.5% with the vaccine, based on a chi-square test with two-sided α = 0.05. Assuming each school will contribute an average of 50 students, and that, from Table 2, the ICC for overall carriage at 12 months is 0.008. The ICC is then used to derive a design effect (DEFF) due to clustering, or variance inflation factor, given by Eq 1:

DEFF=1+(m−1)×ICC, (1)

where m is the average number of participants per cluster. Assuming independent observations, sample size estimates can be multiplied by this DEFF to give the required sample size under a cRCT design. The estimated DEFF in this example is 1.392 (i.e. 1 + (50 − 1) × 0.008). This gives a total required sample size for the cRCT of 1.392×4,644 = 6,465 students per group.

## Discussion

Overall, the ICC for carriage of disease-causing *Nm* genogroups was 0.004 reflecting the low carriage prevalence with little variation observed between metropolitan and rural areas, or on adjustment for other factors. Although a low ICC, this results in a design effect of 1.36. It is notable that in a situation where cluster sizes were larger, this could lead to a larger design effect, further inflating the sample size requirement. Demographically, the cRCT study population was reasonably homogenous and the low ICC for *Nm* carriage indicated that students within schools across the state were only marginally more similar to each other than students from different schools with respect to this outcome. Risk factors that pre-dispose for *Nm* disease and carriage are well established [5] and we expected that within- and between-cluster differences in personal characteristics and behaviours could lead to variability in *Nm* transmission and prevalence, but this did not appear to be the case. It is possible that transmission of *Nm* between students within clusters was contaminated by undifferentiated mixing, i.e. with siblings or others from outside the cluster, or non-participation of some students in the study [22]. Overall, the association between higher prevalence of the characteristic and higher ICC was maintained in the total population and in metropolitan schools. The same association was not necessarily seen in rural schools (e.g. nights out in the past week), although low prevalence characteristics can have and high ICCs and vice versa [23]. There were differences in the degree of correlation for other behaviours in the metropolitan and rural areas. Smoking, for example, was a more correlated behaviour in rural areas compared to metropolitan areas. Such differences may indicate variation in social behaviour at an ecological level in metropolitan and rural areas, e.g. where smoking is a more social behaviour rurally but a more individual pursuit in metropolitan areas. A better understanding of the causal relationship between susceptibility and exposure factors and *Nm* transmission, oropharyngeal carriage and disease, and more complete characterization of social mixing networks would help could help to inform why this might be the case.

This trial was conducted to address a vaccination policy question, in the same population that would be targeted for vaccination in a state program with the already-licensed MenB vaccine. In the absence of baseline or pilot data, baseline carriage prevalence was expected to be low. For the size of the trial required, the cRCT design was optimal to maximize efficiency and operational feasibility, while generating an estimate of total vaccine effect to inform policy decisions. Sample size estimates incorporating an assumed ICC of 0.01 indicated that the total target population was just sufficient to address the research question at a baseline prevalence of 8%. Many of the parameters informing the study design effect were either pre-determined (e.g. enrollment proportion [all schools in the state were invited], mean size of clusters [class sizes within schools were reasonably fixed]), or unknown (no pre-existing data on indirect protective effects of vaccination, nor on the importance of clustering of behaviours and characteristics that pre-dispose to *Nm* carriage in adolescent populations). Had time and resources allowed, baseline data in this age group including detail on social mixing, or simulation where data was available, would have been an advantage. In the final analysis, baseline carriage prevalence was lower than expected, but the ICC observed meant that the validity of the study outcome was largely preserved.

Ultimately, due to the size of the trial and the robustness of its design, the ICCs presented here may be used with confidence by researchers to inform designs where social behaviours in adolescents are important (e.g. when planning cRCTs for *Nm*, *Bordetella pertussis* or pneumococcal carriage), or where social behavioural factors may represent the study outcome in rural or metropolitan or adolescent populations.

## Supporting information

S1 Fig (TIFF)

S1 Table List and definition of risk factor variables included. (DOCX)

## References

[1] Henao-Restrepo AM, Camacho A, Longini IM, et al. Efficacy and effectiveness of an rVSV-vectored vaccine in preventing Ebola virus disease: final results from the Guinea ring vaccination, open-label, cluster-randomised trial (Ebola Ca Suffit!). Lancet (London, England). 2017;389(10068):505–18. doi: 10.1016/s0140-6736(16)32621-6 28017403 PMC5364328

[2] Qadri F, Ali M, Chowdhury F, et al. Feasibility and effectiveness of oral cholera vaccine in an urban endemic setting in Bangladesh: a cluster randomised open-label trial. Lancet (London, England). 2015;386(10001):1362–71. doi: 10.1016/s0140-6736(15)61140-0 26164097

[3] Maiden MC, Ibarz-Pavon AB, Urwin R, et al. Impact of meningococcal serogroup C conjugate vaccines on carriage and herd immunity. The Journal of infectious diseases. 2008;197(5):737–43. 18271745 10.1086/527401 PMC6767871

[4] Peterson ME, Mile R, Li Y, Nair H, Kyaw MH. Meningococcal carriage in high-risk settings: A systematic review. International journal of infectious diseases: IJID: official publication of the International Society for Infectious Diseases. 2018;73:109–17. doi: 10.1016/j.ijid.2018.05.022 29997031

[5] MacLennan J, Kafatos G, Neal K, et al. Social behavior and meningococcal carriage in British teenagers. Emerging infectious diseases. 2006;12(6):950–7. doi: 10.3201/eid1206.051297 16707051 PMC3373034

[6] Tully J, Viner RM, Coen PG, et al. Risk and protective factors for meningococcal disease in adolescents: matched cohort study. BMJ (Clinical research ed.). 2006;332(7539):445–50. doi: 10.1136/bmj.38725.728472.BE 16473859 PMC1382533

[7] Hitchings MDT, Lipsitch M, Wang R, Bellan SE. Competing Effects of Indirect Protection and Clustering on the Power of Cluster-Randomized Controlled Vaccine Trials. American journal of epidemiology. 2018;187(8):1763–71. 29522080 10.1093/aje/kwy047 PMC6070038

[8] Palmu AA, Jokinen J, Nieminen H, et al. Effectiveness of the Ten-valent Pneumococcal Conjugate Vaccine Against Tympanostomy Tube Placements in a Cluster-randomized Trial. The Pediatric infectious disease journal. 2015;34(11):1230–5. doi: 10.1097/INF.0000000000000857 26284652

[9] Zaman K, Sack DA, Neuzil KM, et al. Effectiveness of a live oral human rotavirus vaccine after programmatic introduction in Bangladesh: A cluster-randomized trial. PLoS medicine. 2017;14(4):e1002282. doi: 10.1371/journal.pmed.1002282 28419095 PMC5395158

[10] Roca A, Hill PC, Townend J, et al. Effects of community-wide vaccination with PCV-7 on pneumococcal nasopharyngeal carriage in the Gambia: a cluster-randomized trial. PLoS medicine. 2011;8(10):e1001107. doi: 10.1371/journal.pmed.1001107 22028630 PMC3196470

[11] Lehtinen M, Apter D, Baussano I, et al. Characteristics of a cluster-randomized phase IV human papillomavirus vaccination effectiveness trial. Vaccine. 2015;33(10):1284–90. doi: 10.1016/j.vaccine.2014.12.019 25593103

[12] Murray D.M. and Blitstein J.L. Methods to reduce the impact of intraclass correlation in group-randomized trials. Eval Rev, 2003. 27(1): p. 79–103 doi: 10.1177/0193841X02239019 12568061

[13] ClinicalTrials.gov [Internet]. Bethesda (MD): National Library of Medicine (US). 2000 Feb 29 -. Identifier NCT02849652, Testing an Organizational Change Model to Address Smoking in Mental Healthcare; 2016 Jul 29.[cited 2021 Jun 04]; https://clinicaltrials.gov/ct2/show/NCT02849652

[14] MacIntyre et al. Cluster randomised controlled trial to examine medical mask use as source control for people with respiratory illness. Randomized Controlled Trial BMJ Open. 2016 Dec 30;6(12):e012330. doi: 10.1136/bmjopen-2016-012330 28039289 PMC5223715

[15] Marshall HS, McMillan M, Koehler A, et al. B Part of It protocol: a cluster randomised controlled trial to assess the impact of 4CMenB vaccine on pharyngeal carriage of Neisseria meningitidis in adolescents. BMJ open. 2018;8(7):e020988. doi: 10.1136/bmjopen-2017-020988 29991629 PMC6082482

[16] Marshall HS, McMillan M, Koehler AP, et al. Meningococcal B Vaccine and Meningococcal Carriage in Adolescents in Australia. The New England journal of medicine. 2020;382(4):318–27. doi: 10.1056/NEJMoa1900236 31971677

[17] Australian Curriculum Assessment and Reporting Authority. Guide to understanding ICSEA (Index of Community Socioeducational Advantage) values. Sydney. https://www.myschool.edu.au/more-information/information-for-principals-and-teachers/icsea-for-principals/2015.

[18] Killip S, Mahfoud Z, Pearce K. What is an intracluster correlation coefficient? Crucial concepts for primary care researchers. Ann Fam Med. 2004;2(3):204–8. doi: 10.1370/afm.141 15209195 PMC1466680

[19] Wu S, Crespi CM, Wong WK. Comparison of methods for estimating the intraclass correlation coefficient for binary responses in cancer prevention cluster randomized trials. Contemporary clinical trials. 2012;33(5):869–80. doi: 10.1016/j.cct.2012.05.004 22627076 PMC3426610

[20] Giraudeau B. Model mis-specification and overestimation of the intraclass correlation coefficient in cluster randomized trials. Statistics in medicine. 2006;25(6):957–64. doi: 10.1002/sim.2260 15977300

[21] Macleod CK, Bailey RL, Dejene M, et al. Estimating the Intracluster Correlation Coefficient for the Clinical Sign "Trachomatous Inflammation-Follicular" in Population-Based Trachoma Prevalence Surveys: Results From a Meta-Regression Analysis of 261 Standardized Preintervention Surveys Carried Out in Ethiopia, Mozambique, and Nigeria. American journal of epidemiology. 2020;189(1):68–76. 31509177 10.1093/aje/kwz196 PMC7119302

[22] Leecaster M, Toth DJ, Pettey WB, et al. Estimates of Social Contact in a Middle School Based on Self-Report and Wireless Sensor Data. PloS one. 2016;11(4):e0153690. doi: 10.1371/journal.pone.0153690 27100090 PMC4839567

[23] Gulliford MC, Adams G, Ukoumunne OC, Latinovic R, Chinn S, Campbell MJ. Intraclass correlation coefficient and outcome prevalence are associated in clustered binary data. Journal of clinical epidemiology. 2005;58(3):246–51. doi: 10.1016/j.jclinepi.2004.08.012 15718113

