# Supplementary material for: Intracluster correlation coefficients in a large cluster randomized vaccine trial in schools: Transmission and impact of shared characteristics
Source: PLoS One. 2021 Oct 14;16(10):e0254330. doi: 10.1371/journal.pone.0254330 (PMC8516260; doi:10.1371/journal.pone.0254330)
Supplement: S1 Table — (DOCX) [file pone.0254330.s002.docx]

**S2. List and definition of risk factor variables included.**

| **Variables collected** | **Level** |
| --- | --- |
| **School ICSEA category** | <970, 970-2010, >1020 |
| **School size** | <60 students / year level, 60-119 / year level, >119 students / year level |
| **School location** | Metropolitan, rural |
| **Year of schooling** | Year 10, 11, 12 or 13 |
| **Age** | In years |
| **Gender** | Female / male |
| **Boarding student** | No / Yes |
| **Smoked cigarette in last week** | No / Yes |
| **Smoked water-pipe in last week** | No / Yes |
| **Attending pubs/clubs in the last week** | 0 times, 1 or more times out in last week |
| **Persons kissed intimately in last week** | 0 kissed in the last week, or 1 or more kissed in last week |
| **Current cold or sore throat** | No / Yes |
| **Ethnicity** | White, Aboriginal or Torres Straight Islanders, Asian, Other |

ICSEA: Index of Community Socio-educational Advantage (ICSEA) classification
